# Methotrexate Use in the Management of Ectopic Pregnancies: A Retrospective Study

**DOI:** 10.7759/cureus.95826

**Published:** 2025-10-31

**Authors:** Megan E Slattery, Henry J Nava, Hayley M Spoljaric, Terence D Gipson, Richard Trester

**Affiliations:** 1 Obstetrics and Gynecology, Ross University School of Medicine, Chicago, USA; 2 Obstetrics and Gynecology, University of Los Andes, Merida, VEN; 3 General Surgery, Ross University School of Medicine, Chicago, USA; 4 Health Services Research, Sinai Chicago, Chicago, USA; 5 Obstetrics and Gynecology, Mount Sinai Hospital, Chicago, USA

**Keywords:** barriers to medical management, beta-human chorionic gonadotropin (β-hcg), criteria for methotrexate use, ectopic pregnancy, ectopic pregnancy treated with methotrexate, medical management of ectopic pregnancy, presence of pelvic fluid

## Abstract

Introduction

Ectopic pregnancy (EP), most commonly located in the fallopian tube, presents with symptoms such as abdominal pain, vaginal bleeding, and nausea, which often result in delayed diagnosis, increasing the risk of rupture and life-threatening complications. Methotrexate (MTX) is frequently used to treat stable, unruptured EPs, but success rates vary widely, and optimal dosing strategies and predictive factors remain unclear. This study evaluates MTX efficacy in managing tubal EPs and identifies factors that may guide treatment decisions to improve outcomes and standardize care at an urban academic hospital, with attention to clinical predictors of treatment success and real-world barriers to medical management.

Methods

This retrospective cohort study analyzed cases at Sinai Hospital between June 1, 2019, and May 31, 2024. Thirty-seven women aged 18-45 with confirmed tubal EP were included, encompassing those treated with methotrexate, managed expectantly, or treated surgically. Data collected included initial beta-human chorionic gonadotropin (β-hCG) levels, ultrasound findings, MTX dosing regimen, and serial β-hCG values on days four, seven, and 10. Primary outcomes were time to resolution and need for surgical intervention; secondary outcomes included predictors of MTX failure. Continuous variables were analyzed using t-tests and reported as mean and SD, while categorical variables were evaluated with chi-square or Fisher's exact tests.

Results

Thirty-seven patients (mean age 29.7 ± 5.9 years, gestational age 7.1 ± 2.2 weeks) were analyzed. Initial β-hCG was <1578 mIU/mL in 13 (35%), 1578-4340 in 12 (32%), and >4340 in 12 (32%). MTX success occurred in 9 (24%) patients, most commonly within the 1578-4340 range (n = 7, 58%) and not observed when β-hCG exceeded 4340 mIU/mL. Surgery was required in 22 (60%) patients, predominantly in those with the highest β-hCG. Elevated baseline β-hCG significantly predicted surgical intervention (p = 0.043) and MTX failure; mass size and pelvic free fluid were not significant. The mean surgical length of stay was 1.6 days.

Conclusion

Lower baseline β-hCG levels were the strongest predictor of methotrexate treatment success, whereas elevated β-hCG was significantly associated with treatment failure and the need for surgical intervention. Despite eligibility for medical management, many patients required surgery due to follow-up limitations or institutional preference, underscoring the need for systems that better support outpatient management and optimize patient selections for medical therapy.

## Introduction

Ectopic pregnancy (EP) is an extrauterine pregnancy that most commonly occurs in the fallopian tube. It is an important cause of maternal mortality in the first trimester, accounting for 5-10% of pregnancy-related deaths. The presentation of ectopic pregnancy may be vague and non-specific, with nausea and vomiting; however, typical symptoms include abdominal pain and vaginal bleeding. Women who present with classical symptoms in the first trimester have an 18% prevalence of being diagnosed with an ectopic pregnancy. While about half of EP cases occur in patients without identifiable risk factors, known risks include prior EP, pelvic surgery, fallopian tube damage, infertility, pelvic inflammatory disease, endometriosis, smoking, intrauterine device (IUD) use, and maternal age over 35 years [1,2]. 

In combination with trending beta-human chorionic gonadotropin (β-hCG) levels, other diagnostic measures include transvaginal ultrasound, which can identify both an intrauterine pregnancy as well as an ectopic pregnancy; however, sometimes this method fails to localize the pregnancy, leading to a diagnosis of pregnancy of undetermined location. In 1985, it was well known that at certain β-hCG levels, the pregnancy should be visible on ultrasound, but in the absence of a gestational sac at β-hCG levels greater than 6500 IU/L, this was highly sensitive for the prediction of ectopic pregnancy. Diagnostic laparoscopy is utilized when there is high suspicion, but equivocal ultrasound findings are typically reserved as the gold standard. In some cases of pregnancy of undetermined location, an endometrial biopsy could be performed and further analyzed for the presence or absence of chorionic villi. The presence of chorionic villi is indicative of an intrauterine pregnancy, whereas the absence, in conjunction with a stable β-hCG, suggests ectopic pregnancy. Additionally, dilation and curettage is typically reserved for those with a negative diagnostic laparoscopy work-up when suspicion remains high [3]. Dilation and curettage has shown higher sensitivity for the diagnosis of ectopic pregnancy when compared to endometrial biopsy [2]. 

Delayed diagnosis may result in tubal rupture, leading to internal hemorrhage, hemodynamic instability, and increased maternal mortality. Management options include expectant monitoring, methotrexate (MTX) therapy, or surgical intervention, depending on the patient’s clinical stability and β-hCG trends. Expectant management is considered for asymptomatic patients with low and declining β-hCG levels and no sonographic evidence of a gestational sac. Literature suggests spontaneous resolution in 88% of EPs with β-hCG <200 mIU/mL, and management may be favored in cases with β-hCG <1500 mIU/mL [4]. Given the risk for tubal rupture, patients must be monitored until the β-hCG falls below 15 IU/L, in which most ectopic pregnancies are expected to self-resolve without complications [5-8]. 

Methotrexate, a folic acid antagonist that inhibits DNA synthesis in trophoblastic cells, is generally reserved for hemodynamically stable patients with β-hCG levels <5000 IU/L and adnexal masses ≤5 cm [1,8]. MTX can be administered as a single, double, or multi-dose regimen. Although multi-dose protocols may show higher efficacy, single-dose regimens are associated with fewer adverse side effects and lower costs. A double-dose approach may be considered when β-hCG does not decline by at least 25% within seven days post-treatment. Contraindications to MTX include hemodynamic instability, anemia, immunosuppression, pulmonary disease, renal/hepatic dysfunction, and suspected rupture [2]. Nevertheless, there are no specific guidelines for dosing, predictive factors of success, as well as time to resolution [9].

Surgical management, most often salpingostomy or salpingectomy, is indicated for unstable patients, treatment failure, or contraindications to MTX. Salpingostomy may preserve fertility, but salpingectomy is preferred when the tube is severely damaged, the gestational sac is >5 cm, history of recurrent ectopic pregnancies within the same tube, or bleeding is uncontrolled [10].

Several disparities exist in the management and predictive factors of success in ectopic pregnancies. Methotrexate is most commonly used for the treatment of ectopic pregnancy; however, there is no consensus or protocol when it comes to dosing, which is typically administered as a single dose, double dose, or multiple doses [11]. Methotrexate was first used for the management of choriocarcinoma in 1956, followed by its administration for ectopic pregnancies in the 1960s [12]. The overall success rate with methotrexate use is 75-96% regardless of the method of administration [11], further defined as a 15% decrease in β-hCG levels between days four and seven after treatment [13]. In the literature, it is suggested that a multiple-dose approach may be chosen for higher β-hCG levels, and some studies have suggested that a single dose was associated with higher failure rates; however, other studies found no significant difference. Treatment with multiple doses was indicative of more adverse side effects requiring rescue treatment with leucovorin; however, it posed an overall shorter duration of time for β-hCG levels to fall. Roughly a third of patients receiving a single dose will experience side effects; however, this is much lower than when compared to treatment with multiple doses [11]. The literature cites that double-dose methotrexate was superior to a single dose, with one study finding a 65% success rate and average time of resolution to be 32 days with single dose administration, compared to 93.8% of patients showing a drop of β-hCG by more than 15% among those who received a double dose with average time to resolution to be approximately 58 days [14].

Additional factors contributing to methotrexate dosing include ultrasound findings such as the size of the ectopic pregnancy, fetal heart rate, and the presence or absence of free fluid. Some studies suggest that endometrial stripe thickness may be a predictor of the success of treatment with methotrexate; however, this is not routinely measured. The literature theorizes a direct relationship between endometrial stripe thickness and β-hCG levels; however, it notes that a thickness of 12 mm or greater was associated with a higher failure rate with methotrexate therapy. Most studies cited against the use of methotrexate for ectopic pregnancies greater than 4 cm. The presence of a fetal heart rate was a predictor of methotrexate treatment failure. The finding of free fluid on ultrasound was typically a sign of intraperitoneal hemorrhage, an indicator of tubal rupture, with surgical intervention preferred as opposed to management with methotrexate; however, justification for the use of methotrexate was dependent on the amount of free fluid, as such findings could be physiological; however, the presence of any free fluid was still a risk factor for methotrexate failure [7,8].

This retrospective cohort study aims to investigate the use of methotrexate in ectopic pregnancies at Mount Sinai Hospital in Chicago, IL, to aid in the management of successful treatment modalities and improved patient outcomes, including dosing regimens. This study includes patients who were diagnosed with an ectopic pregnancy from June 1, 2019, to May 31, 2024, and will further dive into treatment decisions, ultrasound findings, and β-hCG levels and trends. The goal of this study is to identify key clinical markers associated with treatment success and to support the development of more consistent treatment guidelines.

## Materials and methods

Study population

This retrospective cohort study was conducted at Sinai Hospital in Chicago, IL, to evaluate the use of methotrexate (MTX) in the management of tubal ectopic pregnancies (EPs) in female patients. The study period spanned from June 1, 2019, to May 31, 2024. Data were extracted from the hospital's electronic medical records for all women, aged 18-45, who were diagnosed with an unruptured tubal EP based on clinical presentation, ultrasound imaging, and elevated β-hCG levels.

The primary analysis focused on patients who received MTX as initial management. Cases managed expectantly or by primary surgery were included descriptively to provide context for real-world treatment selections and to highlight barriers to medical management. Patients were excluded if they had contraindications to MTX, such as hepatic, renal, or hematologic dysfunction, or were outside the study's age criteria. 

Study design

For each patient, data collected included initial β-hCG levels at the time of diagnosis (designated day 0) and relevant ultrasound findings, such as the presence of pelvic free fluid, the size of the ectopic mass, fetal heart activity, and the presence of a yolk sac. The MTX regimen, categorized as either single or double dose, was recorded, along with follow-up β-hCG measurements on days four, seven, and 10 post-treatment. Additionally, the study documented whether surgical intervention was ultimately required, either due to tubal rupture or failed MTX therapy.

The primary outcome of this study was time to resolution, which was defined as the number of days from the initial dose of methotrexate to the resolution of the ectopic pregnancy, determined by the normalization of β-hCG levels or the absence of symptoms. Secondary outcomes included the need for surgical management, in the form of salpingectomy, and the identification of predictive factors for MTX failure.

Statistical analysis

Descriptive statistics were used to summarize patient demographics, clinical characteristics, and treatment outcomes. T-tests were used to examine interactions among all continuous variables, and results are reported as mean ± SD. Categorical variables were analyzed with chi-squared tests and Fisher's exact tests and are expressed as percentages.

## Results

Our study consisted of 37 women, ages 21-41, diagnosed with tubal ectopic pregnancy and treated at Sinai Hospital in Chicago between June 1, 2019, and May 31, 2024. The cohort included patients with confirmed tubal EP who were primarily managed with methotrexate, while those treated surgically or managed expectantly were included descriptively to provide context for overall treatment patterns. Data collected included initial and follow-up β-hCG levels, ultrasound findings, MTX dosing regimen, and whether surgical management was ultimately required. Patients with medical contraindications to MTX (e.g., hepatic or renal dysfunction) were excluded.

Patient characteristics

Thirty-seven women aged 21-41 years (mean 29.73 ± 5.87) were included. The mean gestational age at presentation was 7.06 ± 2.15 weeks, and the mean initial β-hCG was 9871.27 ± 16,376.45 mIU/mL. Mean gravidity was 4.3 ± 2.85, with 2.03 ± 1.55 term births, 1.23 ± 1.63 prior abortions, and 2.33 ± 1.52 living children (Table 1). 

**Table 1:** Demographic and characteristics of patients with tubal ectopic pregnancy (n = 37). β-hCG: beta-human chorionic gonadotropin.

Characteristic	Summary (mean ± SD)
Age	29.73 ± 5.87
Gestational age (weeks)	7.06 ± 2.15
Initial β-hCG	9871.27 ± 16,376.45
Gravida	4.3 ± 2.85
Term births	2.03 ± 1.55
Preterm births	0.07 ± 0.37
History of abortion	1.23 ± 1.63
Living children	2.33 ± 1.52

Baseline clinical findings

Diagnosis and Outcomes

All patients were diagnosed using a combination of serum β-hCG levels and transvaginal ultrasound. Typical findings included adnexal masses and the absence of an intrauterine pregnancy. Of the 37 patients, 11 were successfully treated with methotrexate, three were managed expectantly, and 23 underwent surgical intervention. Among surgical cases, 15 received surgery as the initial management approach, while eight required surgery following failed MTX or expectant management. Surgical cases were included descriptively to illustrate real-world treatment decisions and institutional tendencies, rather than as part of the methotrexate outcome analysis. 

Initial β-hCG was <1578 mIU/mL in 13 patients (35%), 1578-4340 mIU/mL in 12 (32%), and >4340 mIU/mL in 12 (32%). Most ectopic pregnancies were in the right adnexa (51%) or left adnexa (46%). Fetal cardiac activity was present in seven patients (19%), and a yolk sac was visualized in 7 (19%). Mass size was <3.5 cm in 19 patients (51%) and >3.5 cm in 13 (35%), with five cases (14%) undocumented (Table 2).

**Table 2:** Baseline clinical characteristics of patients with tubal ectopic pregnancy (n = 37). β-hCG: beta-human chorionic gonadotropin, EP: ectopic pregnancy.

Variable	Frequency	Percentage
Baseline β-hCG (mIU/mL)		
<1578	13	35%
1578-4340	12	32%
4340-67,170	12	32%
Surgery outcome		
Surgery required	22	60%
Surgery not required	15	40%
Fetal cardiac activity		
No	30	81%
Yes	7	19%
Mass size category		
<3.5 cm	19	51%
>3.5 cm	13	35%
Missing	5	14%
Yolk sac present		
No	29	78%
Yes	7	19%
Missing	1	3%
EP location		
R adnexa	19	51%
L adnexa	17	46%
Missing	1	3%

Management and primary outcomes

**Figure 1:**
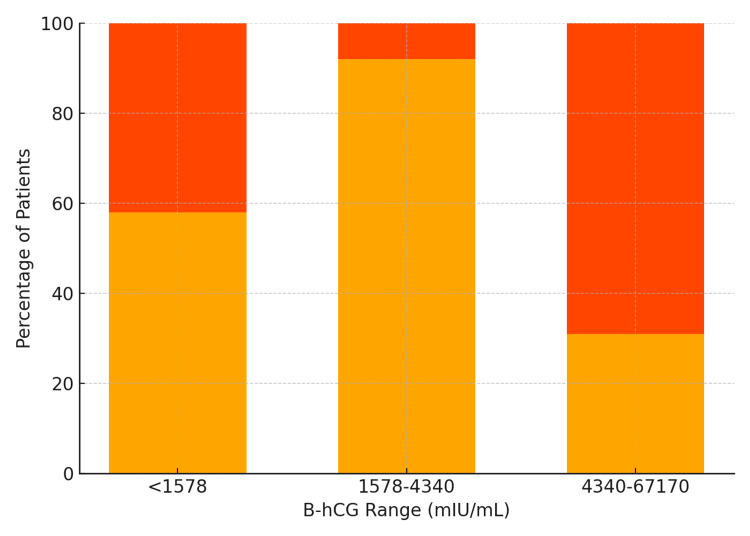
Distribution of initial β-hCG levels across the cohort, stratified by management type (MTX use vs. surgery). Orange: surgery required; yellow: medical management with MTX. β-hCG: beta-human chorionic gonadotropin, MTX: methotrexate.

Of the cohort, 11 patients (30%) had successful methotrexate (MTX) treatment, three (8%) underwent expectant management, and 15 (40%) required surgery as a primary treatment. Patients who were hemodynamically stable and without contraindications were generally considered for MTX therapy. Surgical intervention occurred as initial management in 15 patients, after failed MTX in five patients, and after failed expectant management in three patients (Table 3). All surgical procedures were laparoscopic salpingectomies. When stratified by initial β-hCG, MTX success was most frequent in the 1578-4340 mIU/mL group (7/11, 58%), compared to 3/11 (25%) in the <1578 mIU/mL group and 1/11 (8%) in the >4340 mIU/mL group (Figure 1). The highest surgery rate occurred in the >4340 mIU/mL group (9/13, 69%), which proved to be statistically significant (p-value 0.043) and suggests that β-hCG is a predictor of treatment escalation, as those with higher β-hCG levels were more likely to require surgical intervention. Among surgical patients (n = 23), the mean length of stay was 1.59 ± 0.8 days (median 1.0; range 1-3 days). 

**Table 3:** Treatment outcomes by initial β-hCG level. β-hCG: beta-human chorionic gonadotropin, MTX: methotrexate.

Baseline β-hCG (mIU/mL)	Expectant management (EM)	Failed EM treated by surgery	MTX success	Failed MTX treated by surgery	Treated by surgery	Total
<1578	3 (25.0%)	0 (0.0%)	3 (25.0%)	1 (8.0%)	5 (42.0%)	12 (100.0%)
1578-4340	0 (0.0%)	2 (17.0%)	7 (58%)	2 (17.0%)	1 (8.0%)	12 (100.0%)
4340-67,170	0 (0.0%)	1 (8.0%)	1 (8.0%)	2 (17.0%)	9 (69.0%)	13 (100.0%)
Total	3 (8.0%)	3 (8.0%)	11 (30.0%)	5 (14.0%)	15 (40.0%)	37 (100.0%)

Predictors of surgical intervention

The baseline β-hCG was significantly associated with need for surgery (χ² = 6.31, p = 0.043). Mass size >3.5 cm showed a non-significant trend toward higher surgical rates (p = 0.196) (Table 4).

**Table 4:** Predictors of surgical intervention. β-hCG: beta-human chorionic gonadotropin.

Predictor	Association	Statistical test	p-value
Higher baseline β-hCG	Increased surgery rate	χ² = 6.31	0.043
Mass size >3.5 cm	Non-significant trend	χ²	0.196

Predictors of MTX failure

**Figure 2:**
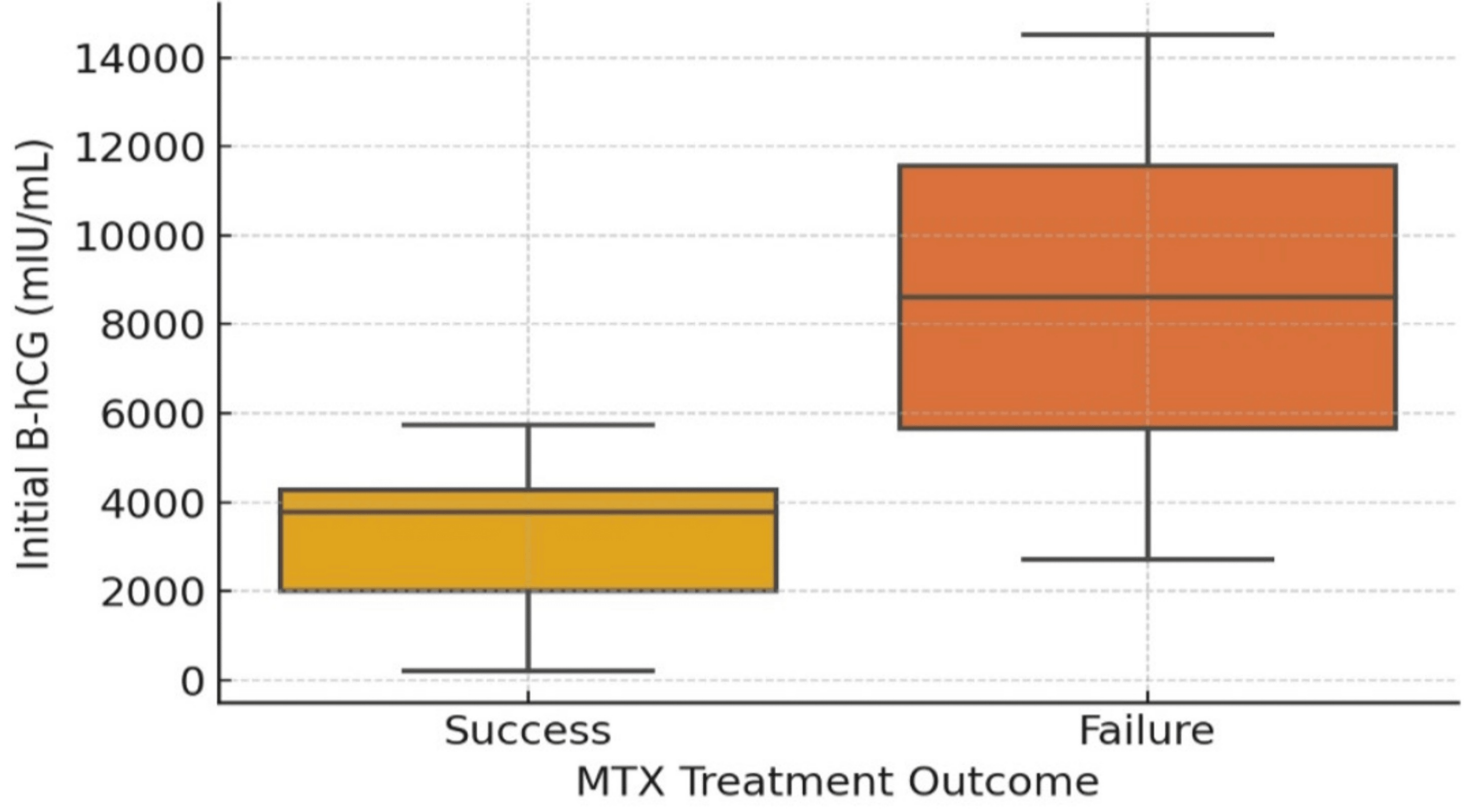
Boxplot comparing baseline β-hCG between MTX success and MTX failure groups, demonstrating higher and right-skewed values in failures. β-hCG: beta-human chorionic gonadotropin, MTX: methotrexate.

**Figure 3:**
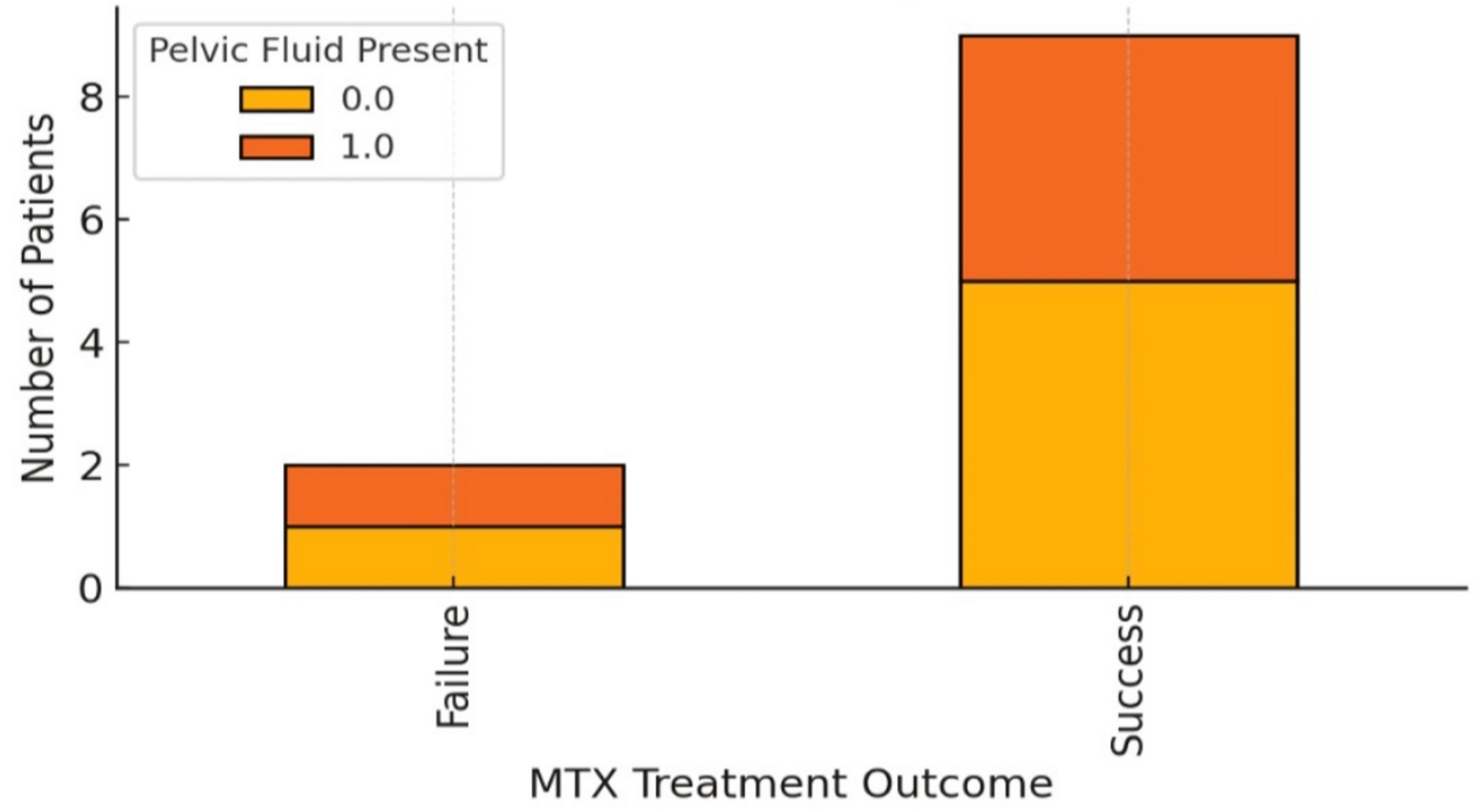
Stacked bar chart showing the presence or absence of pelvic free fluid in MTX success and MTX failure groups. MTX: methotrexate.

Table 5 shows that the representation of patients with MTX failure had higher baseline β-hCG (mean ~8600 mIU/mL) compared with MTX success (mean ~3000 mIU/mL), with a right-skewed distribution in the failure group (Figure 2). The presence of pelvic free fluid was not significantly different between the MTX success and failure groups (Figure 3).

**Table 5:** Predictors of MTX failure. β-hCG: beta-human chorionic gonadotropin, MTX: methotrexate.

Predictor	MTX success (mean ± SD or %)	MTX failure (mean ± SD or %)	Association
Baseline β-hCG (mIU/mL)	~3000	~8600	Higher β-hCG associated with failure
Pelvic free fluid present	Similar between groups	Similar between groups	No significant difference

Psychosocial and structural considerations

While most patients met clinical criteria for MTX, providers often expressed concern over follow-up adherence. Many patients lacked reliable transportation or continuity of care, as evidenced by minimal or absent follow-up β-hCG labs and low outpatient appointment attendance. These barriers, coupled with limited patient understanding of the risks of rupture or need for close monitoring, likely contributed to conservative management choices in favor of surgery.

## Discussion

The goal of this retrospective cohort study was to address key gaps in the medical management of tubal ectopic pregnancies, particularly with respect to the use of methotrexate therapy. Despite its widespread use, there is limited consensus regarding the optimal dosing strategy and reliable predictive factors for successful treatment. Methotrexate use involving single, double, or multiple-dose protocols has been used in the management of hemodynamically stable ectopic pregnancies; however, a consensus on which protocol is optimal has yet to be achieved [5]. Although single-, double-, and multiple-dose methotrexate regimens were used among patients in this cohort, this study was not powered to compare dosing protocols; therefore, dosing approaches are described descriptively in relation to overall treatment outcomes.

This study highlights the nuanced challenges of managing ectopic pregnancies, particularly in underserved populations where follow-up adherence is inconsistent. Despite most patients being clinically eligible for methotrexate based on hemodynamic stability, absence of significant contraindications, and favorable β-hCG levels or ultrasound findings, surgical management was often employed either as the initial treatment strategy or following methotrexate failure. Review of published studies shows that medical management may not always be the optimal choice for a particular patient population, and additional considerations for medical therapy should include patient compliance. One study assessing compliance with MTX therapy found that only 45.5% of patients completed follow-up, only 19.7% completed "appropriate" follow-up, and, of this group, 24% required surgery [15]. These findings underscore a gap between evidence-based guidelines and real-world clinical decision-making, where psychosocial and systemic barriers heavily influence treatment paths.

One of the most striking patterns observed in our data was the relationship between mass size and likelihood of surgical intervention. Patients with adnexal masses greater than 3.5 cm showed a non-significant trend toward higher rates of surgical intervention (p= 0.196), consistent with prior literature suggesting that larger mass size may increase the likelihood of methotrexate failure. In the literature, one article noted that an ectopic mass greater than 3.5 cm was consistent with methotrexate failure, whereas another found that ectopic masses less than or equal to 3.5 cm were optimal candidates for methotrexate therapy (p-value 0.04, odds ratio 2.05) [14,16]. Conversely, patients who experienced successful methotrexate treatment often shared a constellation of favorable features, including lower initial β-hCG levels, smaller mass sizes (<2.0 cm), and an absence of cardiac activity or yolk sac on ultrasound. These clinical characteristics appear to be useful predictors of treatment success and could inform more individualized care protocols. Similarly, failure of methotrexate therapy was linked to higher β-hCG levels and presence of cardiac activity [15].

A critical element in the successful management of ectopic pregnancy is timely and accurate diagnosis, particularly in the emergency department (ED), where most patients in this study initially presented. The diagnostic process for ectopic pregnancy is fraught with challenges, including non-specific symptoms, over-reliance on β-hCG thresholds, and variability in ultrasound interpretation. Delays in diagnosis can significantly increase the risk of morbidity and mortality, especially in cases where rupture has already occurred or is imminent. In our patient population, many individuals presented to the ED with abdominal pain and/or vaginal bleeding, symptoms that warrant immediate transvaginal ultrasound and serial serum β-hCG assessments. However, it is important to emphasize that a reassuring β-hCG level or the absence of classic risk factors should not delay further evaluation. A high index of suspicion must be maintained even when findings are equivocal, as reliance on a single data point may lead to missed or delayed diagnosis.

Emergency physicians play a critical role in the early recognition of ectopic pregnancy and initiation of appropriate management. Importantly, the initiation of methotrexate therapy should not lead to complacency. Patients undergoing medical management still carry a risk of rupture, and a failure to recognize this possibility can result in adverse outcomes. In one retrospective study over a 10-year period that followed 277 women, 41 women, or 15.1% experienced ectopic pregnancy rupture within 25 days of methotrexate treatment [17]. In our review, some patients who met all eligibility criteria for methotrexate later failed treatment and required surgery. These cases underscore the importance of continued vigilance in follow-up, even after treatment has begun.

Beyond diagnostic accuracy, a recurring theme in our data was the profound impact of psychosocial and structural factors on treatment selection and outcomes. Many patients in this cohort demonstrated patterns of delayed or absent follow-up, as evidenced by missed outpatient appointments and incomplete β-hCG trending. Providers frequently cited concerns about a patient’s ability to return for serial monitoring as a reason to pursue surgical rather than medical management. One study discusses the importance of surveillance of β-hCG values following the use of methotrexate, noting that most patients assume the treatment of ectopic pregnancy is complete at the time of injection and do not comply with necessary follow-up. Transportation difficulties, low health literacy, and a general lack of education surrounding the risks of ectopic pregnancy and the importance of follow-up likely contributed to this dynamic. In such contexts, even patients who are ideal candidates for methotrexate from a medical standpoint may face heightened risk if they are not adequately supported throughout the treatment process. One study cited that patients with low socioeconomic status were five times more likely to fail methotrexate treatment. Additionally, a study that looked at undeserved urban populations diagnosed with ectopic pregnancy found that fewer than one in five patients complied with established protocols and more than 50% did not return for follow-up, which can help guide clinicians pick which patients will benefit from medical management, as opposed to surgical management [14].

This study is subject to several limitations, including missing or incomplete data inherent to its retrospective design. The small sample size of 37 patients over the study period reduces statistical power and limits the generalizability of findings. As the data were drawn from a single urban teaching hospital, institutional practices may have influenced management decisions, introducing potential bias and restricting external validity. Methotrexate dosing regimens were not standardized across patients, which makes it difficult to compare treatment protocols or establish regimen-specific outcomes. Certain clinical variables cited in the literature as predictors of methotrexate success, such as endometrial stripe thickness, were inconsistently documented and could not be fully analyzed. Psychosocial and systemic barriers, including transportation challenges, limited health literacy, and inconsistent follow-up, also impacted treatment selection, with some patients being directed to surgery despite meeting criteria for methotrexate. Finally, the lack of reliable follow-up data precluded assessment of long-term fertility outcomes, further limiting conclusions about reproductive impact.

## Conclusions

Methotrexate offers a valuable, fertility-preserving alternative to surgery, but its success depends on careful patient selection and reliable follow-up. In populations at risk for being lost to follow-up, the benefits of medical management must be weighed against the risks of rupture and life-threatening complications. Rather than defaulting to surgical intervention, healthcare systems should invest in infrastructure to support outpatient management, including dedicated follow-up coordination, integration with social work, and culturally tailored patient education.

Understanding predictive factors such as baseline β-hCG levels and adnexal mass size can improve clinical decision-making and help individualize treatment strategies. As found in our study, lower β-hCG levels and a mass size of less than 2.0 cm were predictive factors of methotrexate success and decreased need for surgical intervention. These findings highlight the potential to improve patient outcomes, reduce complications, and establish more standardized guidelines for methotrexate use. Ultimately, the underutilization of methotrexate in certain populations reflects systemic barriers rather than shortcomings of the therapy itself. Improving diagnostic accuracy, patient engagement, and continuity of care may allow more individuals to benefit from fertility-sparing treatments and better align practice with evidence-based standards. 
